# Preferences for mHealth Intervention to Address Mental Health Challenges Among Men Who Have Sex With Men in Nepal: Qualitative Study

**DOI:** 10.2196/56002

**Published:** 2024-03-29

**Authors:** Kamal Gautam, Camille Aguilar, Kiran Paudel, Manisha Dhakal, Jeffrey A Wickersham, Bibhav Acharya, Sabitri Sapkota, Keshab Deuba, Roman Shrestha

**Affiliations:** 1 Department of Allied Health Sciences University of Connecticut Storrs, CT United States; 2 School of Nursing and Health Studies University of Miami Miami, FL United States; 3 Nepal Health Frontiers Tokha-5, Kathmandu Nepal; 4 Blue Diamond Society Dhumbarahi Height, Kathmandu Nepal; 5 Department of Internal Medicine Section of Infectious Diseases Yale School of Medicine New Haven, CT United States; 6 Department of Psychiatry and Behavioral Sciences School of Medicine UCSF Weill Institute for Neurosciences San Francisco, CA United States; 7 Possible Kathmandu Nepal; 8 Department of Global Public Health Karolinska Institutet Stockholm Sweden

**Keywords:** mental health, MSM, mHealth, smartphone apps, digital health, Nepal, gay, homosexual, homosexuality, men who have sex with men, focus group, focus groups, qualitative, barrier, barriers, thematic, mHealth, mobile health, app, apps, applications, applications

## Abstract

**Background:**

Men who have sex with men (MSM) are disproportionately burdened by poor mental health. Despite the increasing burden, evidence-based interventions for MSM are largely nonexistent in Nepal.

**Objective:**

This study explored mental health concerns, contributing factors, barriers to mental health care and support, and preferred interventions to improve access to and use of mental health support services among MSM in Nepal.

**Methods:**

We conducted focus groups with MSM in Kathmandu, Nepal, in January 2023. In total, 28 participants took part in 5 focus group sessions. Participants discussed several topics related to the mental health issues they experienced, factors contributing to these issues, and their suggestions for potential interventions to address existing barriers. The discussions were recorded, transcribed, and analyzed using Dedoose (version 9.0.54; SocioCultural Research Consultants, LLC) software for thematic analysis.

**Results:**

Participants reported substantial mental health problems, including anxiety, depression, suicidal ideation, and behaviors. Contributing factors included family rejection, isolation, bullying, stigma, discrimination, and fear of HIV and other sexually transmitted infections. Barriers to accessing services included cost, lack of lesbian, gay, bisexual, transgender, intersex, queer, and asexual (LGBTIQA+)–friendly providers, and the stigma associated with mental health and sexuality. Participants suggested a smartphone app with features such as a mental health screening tool, digital consultation, helpline number, directory of LGBTIQA+-friendly providers, mental health resources, and a discussion forum for peer support as potential solutions. Participants emphasized the importance of privacy and confidentiality to ensure mobile apps are safe and accessible.

**Conclusions:**

The findings of this study have potential transferability to other low-resource settings facing similar challenges. Intervention developers can use these findings to design tailored mobile apps to facilitate mental health care delivery and support for MSM and other marginalized groups.

## Introduction

Gay, bisexual, and other men who have sex with men (MSM) have poorer mental health and experience more mental distress than their cisgender heterosexual counterparts [1-3]. Studies have shown a high proportion of MSM’s experiences such as mood swings, disordered eating behavior, anxiety disorder, depression, suicidal ideation and behaviors, substance abuse, and body image disorders [4-7]. A recent systematic review and meta-analysis found that the prevalence of depression among MSM in Asia was 37% [6]. These mental health issues experienced by MSM are often linked to stressors triggered by a homophobic environment, particularly due to their sexual orientation [8].

In the context of Nepal, homosexuality is not criminalized, and the rights of MSM are guaranteed by the constitution [9,10]. Despite these legal safeguards, the prevailing cultural norms and societal attitudes pose significant challenges. Traditional and cultural values emphasize heterosexual marriages and family structures and traditional expectations of relationships, and a lack of family support often marginalizes individuals with diverse sexual orientations [11]. These social and cultural characteristics create a heteronormative and stigmatizing environment for MSM, which is detrimental to their mental health. Past studies have found that a very high number of MSM in Nepal had clinically significant depression (54%) and lifetime prevalence of suicidal thoughts (26%) [12,13]. Despite these dire mental health statistics, MSM encounter barriers in accessing health care, particularly mental health services, due to social stigma, discrimination, financial constraints, and insensitivity among health care providers [11,12,14-17]. These barriers to seeking mental health and psychosocial support among MSM, who not only have the highest needs but also the highest unmet needs, give rise to health disparities in this population. In order to reduce these disparities, improving access is crucial for advancing their overall health and well-being.

Mobile health (mHealth), especially mobile apps, offers a promising solution to bridge this gap. It can offer tailored and cost-effective interventions without the need for in-person contact and can provide convenience, improve mental health literacy and easy accessibility, eliminate travel hassles, and encourage help-seeking behavior [18,19]. With Nepal experiencing significant growth in mobile phone ownership of 96% and over 70% using the internet through smartphones, mobile app–based interventions tailored to the needs of MSM in Nepal are potentially feasible [20]. Recognizing the potential of mHealth, we conducted this study to (1) identify the mental health challenges and barriers to accessing mental health and psychosocial support services among MSM and (2) understand their preferences for smartphone apps (eg, functionality, format, design, and attributes) that could enable their access to mental health and psychosocial support services access.

## Methods

### Study Setting and Recruitment

This qualitative study is part of a larger HIV biobehavioral survey that was conducted among 250 MSM participants in Kathmandu, Nepal [13]. Five focus group (FG) sessions were conducted with MSM participants in January 2023. Four of these sessions included 6 MSM participants in each, while the remaining session had 4 participants (N=28). FG sessions were conducted until a point of theoretical saturation was achieved. Eligibility criteria for participation included: (1) 18 years or older, (2) self-identified as cisgender MSM, and (3) proficiency in Nepali or English.

Participants were recruited using respondent-driven sampling, a network-based sampling method often used for hard-to-reach populations. The recruitment chain was initiated with 5 MSM “seeds,” purposively selected based on recommendations from a community-based organization providing services to MSM. Each seed who completed the interviewer-administered questionnaire was given 5 recruitment coupons to recruit potential peers. Subsequent participants were, in turn, given 5 coupons to recruit additional peers. In total, 28 (∼11%) of the survey participants were randomly selected for the FG sessions.

### Study Procedure

FG sessions were conducted inside the community-based organization’s office and lasted about 90 minutes. A semistructured FG topic guide with appropriate probes was developed that guided the discussion. A trained facilitator led the FG sessions, and a cofacilitator took the notes. Both the facilitator and cofacilitator identified themselves as MSM. 

Before the discussion, participants completed an interviewer-administered Qualtrics survey that included sociodemographic, sexual health, alcohol, smoking, violence, and mental health–related questions. The participants’ exposure to violence was assessed using the 4-item Hurt, Insult, Threat, and Scream screening tool, using a 5-point frequency format (scores 4-20). Final scores were classified as normal (0-10) or violence (11-25) [21]. Depressive symptoms were evaluated with the Patient Health Questionnaire instrument, scoring each of the 9 *Diagnostic and Statistical Manual of Mental Disorders*, 4th edition, criteria (0-3). A composite score of 0-27 was computed, with a score exceeding 10 indicating moderate to severe depressive symptoms [22].

The FGs involved questions and discussions about traumatic life events. Participants were made aware that they did not have to answer any questions that they felt were distressing and could leave the FG session at any time if they felt uncomfortable. A study team member was also present at all 5 FG sessions to refer to a counselor or provide any additional support needed in the case of a distressing situation. While conducting the FG sessions, a trained facilitator approached participants sensitively, respecting moments of silence and their willingness to continue discussions—statements like “I am fine” or “we can continue” followed silence. Despite the sensitive topics discussed, none of the participants requested support, including speaking with counselors. At the end of all FG sessions, participants also disclosed that they were glad to have had the opportunity to share their experiences.

### Data Analysis

SPSS (version 29.0.0 software; IBM Corp) was used to calculate descriptive statistics (frequencies and percentages) for the variables collected via a Qualtrics survey. FG transcripts were transcribed and checked for accuracy before coding. The 2 coders (KG and CA) read and reread transcriptions to identify key ideas and recurring themes. A codebook was developed with mutually agreed-upon codes derived from the FG transcripts, and coding was completed independently by 2 researchers (KG and CA). To ensure reliability, codes were constantly compared for agreement and discussed between the coders, and the senior author (RS) cross-checked all codes. Dedoose (version 9.0.54) was used for data management and analysis. The themes were gathered as child codes and then placed into a broad category as root codes. Each theme with its qualitative quotes to best illustrate the findings are presented in the results section.

### Ethical Considerations

The study protocol was approved by the institutional review boards at the University of Connecticut (H22-0039) and the Nepal Health Research Council (2391-2022 P). All the participants provided verbal informed consent before their participation. Participants were explained the importance of maintaining the confidentiality of FGs and requested not to discuss the experiences and comments shared during the FGs with others. All the sessions were conducted in Nepali and were audio recorded, transcribed, and translated. Participants were compensated NRs 1000 (~US $8) for their time and participation. FG transcripts were deidentified before the analysis, and the survey data were anonymous.

## Results

### Participant Characteristics

Table 1 provides information on participants’ characteristics. The mean age of study participants was 25.3 (SD 6.1) years. Most of the 28 participants were Hindu (n=22, 79%), had a high school or higher degree (n=21, 75%), and identified as gay (n=22, 79%). A total of 21% (n=6) of participants had depressive symptoms, and 14% (n=4) had experienced violence in their life. A little over half (n=15, 54%) of participants had used health-related mobile apps, and almost 90% (n=25) used digital devices to search for health-related information.

**Table 1:** Participant characteristics (N=28).

Sociodemographic factors			Values
Age (years), mean (SD)			25.3 (6.1)
**Religion, n (%)**			
	Hindu	22 (79)	
	Buddhist	5 (18)	
	Others	1 (4)	
**Level of education, n (%)**			
	Up to grade 10	7 (25)	
	High school and above	21 (75)	
**Employment, n (%)**			
	No	15 (54)	
	Yes	13 (46)	
**Income level, n (%)**			
	Less than NRs 20,000 (∼US $150)	12 (43)	
	NRs 20,000 (∼US $150) and above	16 (57)	
**Sexual orientation, n (%)**			
	Gay	22 (77)	
	Bisexual	6 (21)	
**Relationship status, n (%)**			
	Single	19 (68)	
	With partner	9 (32)	
**Depressive symptoms, n (%)**			
	No	22 (79)	
	Yes	6 (21)	
**Ever experienced violence, n (%)**			
	No	24 (86)	
	Yes	4 (14)	
**Daily smoker, n (%)**			
	No	5 (18)	
	Yes	23 (82)	
**Alcohol use (past 12 months), n (%)**			
	No	6 (21)	
	Yes	12 (79)	
**HIV status, n (%)**			
	Positive	1 (4)	
	Negative	27 (96)	
**Syphilis status, n (%)**			
	Positive	8 (29)	
	Negative	20 (71)	
**Engaged in anal sex (past 6 months), n (%)**			
	No	6 (21)	
	Yes	22 (79)	
**Condomless sex (past 6 months), n (%)**			
	No	11 (39)	
	Yes	17 (61)	
**Sexual partners in (past 6 months), n (%)**			
	Single	17 (61)	
	Multiple	11 (39)	
**Engagement in group sex (past 6 months), n (%)**			
	No	26 (93)	
	Yes	2 (7)	
**Engagement in sex work (past 6 months), n (%)**			
	No	26 (93)	
	Yes	2 (7)	
**Has any health insurance, n (%)**			
	No	24 (86)	
	Yes	4 (14)	
**Use of health-related apps in mobile, n (%)**			
	No	13 (46)	
	Yes	15 (54)	
**Use of mobile or technological devices to search for health-related information, n (%)**			
	No	3 (11)	
	Yes	25 (89)	

### FG Results

#### Overview

Throughout the data analysis, 3 overarching themes emerged in the codebook with their own subthemes (Multimedia Appendix 1): (1) mental health challenges, (2) barriers to accessing mental health services, and (3) preference for mental health mobile apps with desired features and attributes.

#### Mental Health Challenges

Mental health challenges faced by the participants involve a multifaceted interaction of factors, including sexual orientation, emotional distress, stigma, discrimination and victimization, and social exclusion. Moreover, they frequently encounter barriers to accessing support services that could enhance their mental well-being. Participants not only vividly described their day-to-day challenges but also shared insights into the collective experiences of the lesbian, gay, bisexual, transgender, intersex, queer, and asexual (LGBTIQA+) community. Their comprehensive perspective underscored the profound impact of prevailing societal biases on their mental well-being (Textbox 1).

A constant fear of societal judgment and family pressure to conform to traditional gender norms has intensified issues like anxiety, depression, and suicidal thoughts. Participants in all FGs highlighted the pressure to enter heterosexual marriages, causing emotional turmoil as they navigate their identities and societal expectations (Textbox 2).

Participants disclosed coping mechanisms, such as drug use, drinking alcohol, smoking, engaging in sexual risk behaviors (eg, multiple sex partners), and self-harm. These strategies were described as providing temporary relief from the immense emotional turmoil they experience.

I have personally known someone who started risky sexual behavior from a young age because that was how they felt validated. They wanted others to make them feel better. So, they would often engage in multiple sexual encounters, thinking it would help them cope with their struggles... I also know people who turned to drugs and alcohol to cope with themselves.

18-year-old participant from FG1

…due to tension and mental pressure, it was tough for me to control myself, so I started to cut my hands with a razor; I did it many times. I was also thinking of taking tablets for suicide.

30-year-old participant from FG3

Several participants talked about and shared their experiences of intense anxiety and fear surrounding the possibility of contracting HIV and other sexually transmitted infections following sexual encounters with their partners.

I have extreme fear about whether I contracted it [HIV] or not… even the close friends I know have contracted HIV, and because of that, I also have a fear and anxiety of whether I contracted HIV or not after the sex is done.

21-year-oldparticipant from FG1

**Social acceptance and lack of family support heighten mental health challenges.**

- “…there is no one, and when we open up there is no family support. Family supporting queer people, it is like gold which is rare. We only open out in this [LGBTIQA+] community; You can imagine how bad is our mental status and the situation.” [26-year-old participant from FG2]
- “…because of my sexuality, sometimes I suffer from social anxiety, ‘are they judging me because of my looks, voice or the way I dress.” [24-years-old participant from FG5]
- “I study in 12th grade, and most of the time, I am bullied by my male classmates… even the teachers ask, “Why do you act like a girl?” And most of them do know I use TikTok, and everyone knows about me, so I think bullying is also another part, and I think mental health or stress is a common occurrence for everyone in LGBTIQA+ people.” [22-year-old participant from FG1]

**Mental health challenges from societal pressure and identity concealment.**

- “…if I have to be ‘me’ or do something feminine, then there is a fear of being judged by other people, so I have to pretend as a closeted man, I have to pretend masculine, have a masculine voice, and all the stereotypes and stigmas the people in the community have kept, which fuels the anxiety.” [22-year-old participant from FG1]
- “I faced immense pressure from my family about marriage, being the only son. I kept my sexual orientation hidden, making it harder in our community. The talks of marriage became unbearable… I felt so distressed that I left home and was even suicidal. My relatives found out, but the misunderstanding about my identity remained... these struggles took a toll on my mental health, forcing me to search for ways to cope and maintain my well-being.” [28-year-old participant from FG3]
- “I was so low that all I had in mind was suicide.” [29-year-old participant from FG2]

Participants also shared that they fear the potential disclosure of their HIV status because they anticipate that others may treat them differently after learning their status.

When it comes to HIV, if a person status has HIV, he is afraid that if his status leaks, then people will look differently.

23-year-old participant from FG4

One participant recounted how their colleague, upon learning their HIV-positive status, tragically died by suicide, underscoring the emotional toll and mental health challenges.

There was one colleague of mine who died by suicide as soon as the HIV test result came back positive.

25-year-old participant from FG1

#### Barriers to Accessing Mental Health Services

Participants shared that many gay, bisexual, and MSM do not seek mental health services because they perceive themselves as mentally healthy and believe their lives are going well, leading them to overlook the need for such support.

The reason I believe that our community members do not seek mental health services is because they think they are alright, that their life is just going on, they think they are fine and healthy and feel they don’t need such services.

25-year-old participant from FG3

Participants also shared that individuals tend to become more open and willing to seek help if they are aware of mental health services like counseling and therapy.

…if people are aware of counseling and therapy, people will be more willing to go there.

24-year-old participant from FG2

Participants discussed that individuals still closeted about their identity find it challenging to trust others, creating a communication barrier. Their hesitancy to trust stems from a history of hiding aspects of themselves, hindering open communication and sharing true feelings and experiences.

It is hard for people to trust. There is also a communication barrier because they are still closeted and grew up hiding things from the beginning. If the person themselves is not trusting them, then how can they trust the person in front of them.

35-year-old participant from FG2

Stigma and discrimination associated with mental health and sexuality were major concerns for participants. Many participants brought up fears of being labeled “*pagal*” (a pejorative that is closest to “crazy” in English) as a barrier to accessing mental health services.

…there is a stigma against mental health, that is the reason we do not seek mental health services. If we visit a health care center, then people will talk about it, and the peer groups and society will think of us as pagal (crazy); they will say that this person is taking medicines, so that is another reason we do not visit mental health care centers.

26-year-old participant from FG3

Others discussed the impact that homophobia can have on MSM seeking mental health services. Homophobia and heterosexism still exist in Nepal’s society and can have significant impacts on MSM decisions.

A stereotypical saying “how can men like men?” is still prevalent in society, so, to not get judged by others, people don’t attend these [mental health] sessions.

29-year-old participant from FG2

Many participants expressed their frustration with medical professionals who, instead of addressing their health concerns seriously, tend to label them, dismiss their issues, and attribute symptoms to perceived psychological factors such as overthinking, thereby hindering their access to necessary services.

Often, the doctor calls us with names, gives us a tag, they do not give us a priority, they only say “there’s nothing wrong with you, it’s only because you overthink” which has an impact on seeking services.

26-year-old participant from FG3

Many participants expressed the financial strain posed by mental health services for MSM in Nepal. The consensus was that the cost associated with psychiatrist visits, along with their limited financial resources, significantly affects MSMs’ ability to access the necessary mental health services.

The main reason is finances because it is still very expensive, like we have to pay NRs 800 to 1000 (approximately US $8 to 10) per visit. It is expensive, even more so in private clinics.

23-year-old participant from FG5

Several participants across all FG sessions expressed concerns about time limitations and transportation challenges when it came to accessing mental health services. In an FG, a majority of participants agreed on the considerable difficulty that MSM faces in securing transportation to be able to go to a physical mental health appointment. In another session, everyone unanimously agreed and nodded in agreement with the following statement:

I think so too, because not everywhere has access to transportation, and for some places, we might even have to walk a lot to reach there.

27-year-old participant from FG2

When it comes to time constraints, participants talked about the difficulty of scheduling mental health appointments within the confines of work or school hours. They highlighted the difficulty of taking leave from work or school to attend counseling sessions during times of need.

I can go frequently, but the counseling appointment has to be time-friendly. Some of us are employed from 10 am to 5 pm or even 6 pm. If the counseling session is around that time, then I might come for a few days, taking a leave from work, but if my office does not allow me, then even if I had a mental health support need, I would not be able to attend.

26-year-old participant from FG3

#### Solution: Mental Health Smartphone Apps With Desired Features and Attributes

##### Overview

Participants expressed a preference for a smartphone app with a variety of features and attributes compared to traditional clinical settings. They foresaw that such an app could enhance understanding of mental health, offer convenience, improve accessibility, reduce the necessity for travel and associated expenses, and deliver services in a confidential and nonjudgmental setting.

During our young age, we didn’t have any type of apps to help with our issues or any sort of networking apps like Grindr, but now people are more open to using apps, so creating an app to help solve the mental health issue and counsel can be a great idea.

35-year-old participant from FG2

##### Desired Features of the Mobile App

Participants recommended using creative approaches, such as fun activities to assess individuals’ mental health for early detection, moving away from more direct approaches.

Something creative, not a direct approach, but through games or other ways we could assess the mental health status of the people for early detection.

25-year-old participant from FG3

Participants emphasized the importance of using the app to schedule regular counseling appointments with mental health professionals for those requiring assistance. There was a strong preference for using Zoom over platforms like Viber and WhatsApp for digital counseling, citing its widespread use during the COVID-19 pandemic.

…those who are in need of mental health services should get counseling appointments from a professional by selecting them once or twice a week in the app.

29-year-old participants from FG4

Rather than Viber and WhatsApp, Zoom is good for e-counseling, as in COVID many people are using it.

18-year-old participants from FG4

All participants underscored the importance of mental health and psychosocial service providers being qualified, friendly, and supportive of the LGBTIQA+ community. They stressed the need for an environment where individuals feel safe and comfortable to share their concerns.

First of all, they should be very friendly towards LGBTIQA+, no matter whether they are a community member or not, and we have to feel safe and able to share everything. Is qualified and has studied the related field.

23-year-old participant from FG5

Some participants had suggestions that would help make MSM more comfortable in participating in digital counseling, such as making cameras not compulsory.

We can do it through audio calls. Zoom counseling sessions are fine, but opening cameras should not be necessary or compulsory.

21-year-old participant from FG5

An additional recommendation included providing convenient hours, allowing users of the app to secure digital counseling appointments relatively quickly. This would accommodate individuals who work or go to school, ensuring continued accessibility to the services.

People will schedule according to their needs and how big their problem is, if you are having a problem now and get an appointment for a session after a month, it is not possible.

21-year-old participant from FG2

Participants suggested incorporating a toll-free helpline number within the smartphone app. They shared their experiences with toll-free helplines that did not function as intended in the past. Additionally, they provided suggestions for improving the toll-free helplines within the mobile app.

We can use a Toll-free helpline number, but even I tried to use toll-free service every time it was busy. So, the missed call system [call back system] is good.

21-year-old participant from FG5

Several participants suggested including a feature to message counselors in addition to the toll-free helpline that could help those who do not want to or cannot talk over the phone.

Some might not want to speak; they could talk through chat.

30-year-old participant FG3

Several participants shared their difficulties in finding friendly mental health and psychosocial service providers. To address this issue, participants suggested having a directory of LGBTIQA+-friendly providers on a mobile app that would help show MSM where to go when they require help.

I searched, and I came to know. It took me a lot of effort, and it was hard to find psychosocial counselors.

29-year-old participant from FG2

Participants also suggested to include mental health educational resources, especially in the form of videos.

Many are hidden, they do not even want to come out of the house, because of the fear of society. But they use mobile apps, they could connect to the app, and even with information and educational videos, we could reach them.

21-year-old participant from FG5

Many participants suggested a feature to connect with peers and other members of the MSM community through a communication channel within the app. They highlighted the importance of such a platform for sharing experiences and emphasized the value of peer support.

I think a discussion forum would be a good addition. The forum can help you share and make you feel like you are not the only one who is going through the same trauma and hardships, and we will be sharing with each other.

21-year-old participant from FG1

##### Attributes of the App

Many participants suggested placing special emphasis on the privacy and confidentiality of data collected by the app. They recommended that app developers and health care providers should commit to privacy and confidentiality clauses in their contracts, with strict consequences for any breaches of information.

The staff, app developers, and providers should sign on privacy and confidentiality in their contract. If leakage of information is found, they need to know that strong steps will be taken.

26-year-old participant from FG3

When discussing the user interface and colors of the app, several participants suggested that the mobile app should not overtly appear targeted exclusively at the LGBTIQA+ community. The participant expresses a desire for the app to have a discreet appearance, in contrast to the distinctiveness of dating apps targeted toward LGBTIQA+.

Through application maybe, the application should not look like for only LGBTIQ. It must look normal, not like Grindr.

23-year-old participant from FG5

Participants showed a strong interest in an engaging activity for user engagement and retention, particularly one that incorporates entertainment. One participant mentioned:

There has to be an environment in the mobile app so that I feel like going and using it again.

26-year-old participant from FG3

## Discussion

### Principal Findings

This study revealed a complex interplay between mental health challenges, including depression, anxiety, and suicidal behavior, among MSM in Nepal. The findings further highlight the barriers to accessing mental health care and support services among Nepali MSM due to factors such as insufficient mental health literacy, privacy concerns, financial strain, stigma, and discrimination. This underscores the urgent need for tailored and accessible mental health interventions. Participants overwhelmingly preferred smartphone app interventions to address the identified barriers and challenges, emphasizing their preference for accessible and confidential mental health support through digital platforms.

The major concern among MSM, where individuals perceive themselves as “all right” without the need for mental health services and less help-seeking attitudes, likely indicates a lack of mental health literacy, which is similar to the findings from studies among men and other minority populations [23-27]. Participants in this study expressed a preference for mental health resources and screening tools integrated into the app. Few studies have demonstrated that a smartphone app with an easily accessible and comprehensive mental health education module, resources, and engaging screening tools has the potential to combat this issue by fostering a proactive attitude toward mental well-being, the importance of seeking support, and the early detection of mental health problems [28-30].

The stigma and discrimination faced by MSM, both within society and health care settings, contribute to hesitancy in seeking mental health support. This fear of stigma and reluctance aligns with the findings from studies of various marginalized populations [26,31-33]. In response to this, participants expressed a preference for features within the mobile app that could link participants with LGBTIQA+-friendly mental health professionals through video sessions, automated text messages, or phone calls, emphasizing the crucial role of trust and understanding in the provider-patient relationship. Few interventions have integrated such features into digital interventions [34,35]. This feature could help to overcome this barrier by connecting individuals with LGBTIQA+-friendly and supportive mental health professionals and fostering a more inclusive, judgment-free, and accessible mental health support system.

In line with a substantial body of research, the findings emphasize that various stressors, particularly those related to societal biases, discrimination, fear of HIV, and other sexually transmitted infection results, contribute to psychological distress, and these influence maladaptive coping behaviors among MSM [7,8,36-38]. By incorporating features such as mental health resources, coping strategies, and peer support discussion forums, the app can have the potential to empower MSM to navigate these challenges more effectively.

The privacy and confidentiality concerns expressed by MSM underscore the need for a sensitive approach to mental health support. This apprehension aligns with findings from studies of various minority populations [39-42]. Participants in this study articulated the desire for a mobile app that explicitly addresses these concerns through robust consent forms, privacy features, and secure messaging platforms. The app could have features that aim to ensure privacy and confidentiality, potentially fostering MSM trust and addressing barriers related to sharing personal mental health information. Integrating these features into app design could significantly contribute to alleviating privacy concerns and establishing a secure environment that encourages seeking mental health support.

The cost of accessing mental health services was a major concern for participants in the study, which aligns with previous research on the cost of mental health in Nepal [43]. It is important to address financial strain in any intervention that is created to help MSM in Nepal with mental health [44-46]. Studies have found that, by reducing travel expenses, mHealth interventions help allow access for sexual minority individuals to mental health care [47,48]. This not only addresses the financial challenges faced by Nepali MSM but also alleviates the transportation struggles [35].

### Strengths and Limitations

This study is of particular value due to the lack of participant involvement in the development of mental health interventions, with LGBTIQA+ consultation being notably rare when it comes to the creation of health interventions, policies, or guidelines [49,50]. Using FGs, the participants’ perspectives can be used to create a more tailored and effective digital health intervention. However, this study has its own limitations. One of these limitations is the presence of social desirability bias, which is a common occurrence in FG discussions. This bias can influence participants to express socially acceptable opinions rather than their true thoughts and feelings. Additionally, it is worth noting that the study was done in Kathmandu, Nepal, which can differ in culture and access to mental health services than other areas of Nepal, limiting the transferability of the study findings mainly on the challenges and barriers. Finally, it is important to consider that the desire to participate in a given intervention does not automatically guarantee its real-world adoption. Evaluating the actual usage and effectiveness of the intervention in real-life scenarios is crucial to fully understand its impact and potential benefits. Therefore, it is necessary to evaluate real-world usage.

### Conclusions

The study highlights the mental health challenges encountered by MSM in Nepal and the barriers they face in accessing mental health support services. The participants’ direct quote, “invisible in the corner of the room,” captures the hidden nature of their struggles intimately tied to the intersectional stigma surrounding mental health and sexuality. Emphasizing the potential of mobile apps, our findings suggest that incorporating user-friendly features like accessible resources, mental health screening tools, and digital counseling with LGBTIQA+-friendly providers can bring visibility to the mental health challenges of MSM. The mobile app has the ability to establish an open and supportive space, breaking down barriers and offering a pathway for MSM in Nepal to identify and address their mental health concerns with ease and confidence.

## Appendix

Multimedia Appendix 1 Parent and child codes with description.

## Abbreviations

FG: focus group
mHealth: mobile health
MSM: men who have sex with men
LGBTIQA+: lesbian, gay, bisexual, transgender, intersex, queer, and asexual
