# Supplementary material for: Preferences for mHealth Intervention to Address Mental Health Challenges Among Men Who Have Sex With Men in Nepal: Qualitative Study
Source: JMIR Hum Factors. 2024 Mar 29;11:e56002. doi: 10.2196/56002 (PMC11015371; doi:10.2196/56002)
Supplement: Multimedia Appendix 1 [file humanfactors_v11i1e56002_app1.docx]

**Table S1.** Root and child codes with description.

| **Root Code** | **Child Code** | **Description of Code** |
| --- | --- | --- |
| **Mental Health Challenges** | Lack of Family Support | The lack of familial understanding contributes to heightened mental stress. |
|  | Societal Biases and Bullying | Biases, stereotypes, and bullying were described as mentally torturous, having lasting effects on the mental well-being. |
|  | Expectation of Heterosexual Marriages | Struggles with the societal expectation to enter heterosexual marriages and conform to traditional gender norms leading to anxiety, depression, and suicidal tendencies |
|  | Stress Coping Mechanisms | Stress coping mechanisms such as drug use, alcohol consumption, smoking, risky sexual behaviors, and self-harm, including thoughts of suicide, as a temporary relief from the emotional turmoil. |
|  | Fear of HIV and STIs | Anxiety and fear surrounded the possibility of contracting HIV and other STIs following sexual encounters. |
|  | Fear of HIV Status Disclosure | Fear of the potential disclosure of HIV status and its consequences. |
|  | HIV Positive Results | Instances where individuals, upon receiving a positive HIV test result, exhibit suicidal tendencies |
| **Barriers to Accessing Mental Health Services** | Lack of Awareness | Limited awareness about mental health services. |
|  | Privacy Concerns | Privacy and confidentiality concerns when using mental health services, including lack of trust between users and providers. |
|  | Intersectional Stigma | The stigma associated with mental health and sexuality impacting the decisions to access mental health services. |
|  | Discrimination | The discrimination and stereotyping from society, including healthcare providers, based on sexual orientation. |
|  | Lack of LGBTIQA+ friendly Providers | The absence of LGBTIQA+ friendly providers contributing to a lack of trust in seeking mental health services. |
|  | Financial Strain | The cost of mental health services and limited financial resources to access necessary services. |
|  | Accessibility Issues | Concerns about time constraints and transportation options making it difficult for MSM to access mental health services. |
| **Mental Health Interventions** | App Preferences | Preference for mobile app interventions over traditional clinical settings. |
| **Preferred Features in the App** | Mental Health Screening | Suggestions include creative methods for mental health screening in the mobile app. |
|  | Online Counseling | Participants preference to include a feature to connect with providers by booking appointments via an app and connecting virtually. |
|  | LGBTIQA+ Friendly Providers | A strong emphasis on having mental health service providers who are friendly, qualified, and supportive of the LGBTIQA+ community. A safe and comfortable environment is deemed essential for sharing concerns. |
|  | Toll-Free Helpline | Participants suggestion for the inclusion of a toll-free helpline in the app for immediate mental health support |
|  | Messaging and Chat Features | Participants suggestion for the inclusion of messaging and chat features to communicate with counselors through chat for those who prefer communication over toll-free phone calls. |
|  | LGBTIQA+ Friendly Directory | A directory of LGBTQA+ friendly mental health service providers within the app to assist MSM in finding supportive resources when needed. |
|  | Educational Resources | Participants suggestion for the inclusion of mental health educational resources, particularly in video format. |
|  | Discussion Forum | Participants recommendation to include a communication channel within the app for connecting with peers, such as a discussion forum for sharing experiences and providing peer support. |
| **Preferred Attributes of the App** | Privacy and Confidentiality Commitment | Participants recommendation for strict contractual commitments to uphold privacy and confidentiality during the app’s development and implementation, with severe consequences for information leaks |
|  | Inclusive Design | Suggestions for the app’s user interface include a discreet appearance to avoid exclusivity, unlike LGBTIQA+ dating apps |
